# Cytotoxic and mutagenic effects of the food additive tartrazine on eukaryotic cells

**DOI:** 10.1186/s40360-022-00638-7

**Published:** 2022-12-23

**Authors:** Jailson Rodrigues dos Santos, Larissa de Sousa Soares, Bruno Moreira Soares, Marlene de Gomes Farias, Victor Alves de Oliveira, Natan Antônio Batista de Sousa, Helber Alves Negreiros, Felipe Cavalcanti Carneiro da Silva, Ana Paula Peron, Ana Carolina Landim Pacheco, Márcia Maria Mendes Marques, Juan Carlos Ramos Gonçalves, Raquel Carvalho Montenegro, Muhammad Torequl Islam, Javad Sharifi-Rad, Mohammad S. Mubarak, Ana Amélia Carvalho de Melo Cavalcante, João Marcelo de Castro e Sousa

**Affiliations:** 1 Laboratory of Cytogenetics and Mutagenesis of the Federal University of Piauí, Picos, Brazil; 2 Laboratory of Human Cytogenetics and Oncology Research Center, Federal University of Pará, Belém, Brazil; 3 Cytogenetic and Mutagenesis Laboratory, Postgraduate Program in Pharmaceutical Sciences, Federal University of Piauí, Teresina, Brazil; 4 Cytogenetic and Mutagenesis Laboratory, Postgraduate Program in Genetics and Improvement of the Federal University of Piauí, Teresina, Brazil; 5 Laboratory of Parasitology and ecology of neglected diseases, Federal University of Piaui, Terezina, Brazil; 6 Department of Biochemistry and Pharmacology, Federal University of Piaui, Terezina, Brazil; 7 Drug Development and Research Center, Federal University of Ceara, Fortaleza, Brazil; 8 Department of Pharmacy, Life Science Faculty, Bangabandhu Sheikh Mujibur Rahman Science and Technology University, Gopalganj8100, Dhaka, Bangladesh; 9 Facultad de Medicina, Universidad del Azuay, Cuenca, Ecuador; 10 Department of Chemistry, The University of Jordan, Amman, 11942 Jordan

**Keywords:** Carcinogenesis, Tartrazine, Cell line, *Allium cepa*, *Artemia salina*, *Saccharomyces cerevisiae*

## Abstract

**Background:**

Among the food additives used in the food industry, food dyes are considered the most toxic. For instance, tartrazine (TRZ) is a food colorant commercially available with conflicting data regarding its cytotoxic, genotoxic, and mutagenic effects. Therefore, this study aimed to evaluate the cytotoxic and mutagenic potential of TRZ using different eukaryotic cells (*in vitro*).

**Methods:**

This study employed 3-(4,5-dimethyl-2-thiazolyl)-2,5-diphenyl-2H-tetrazolium bromide (MTT), brine shrimp lethality, *Allium cepa* and *Saccharomyces cerevisiae* tests. Different concentrations of TRZ and different exposure times were used in this study.

**Results:**

The results demonstrate that TRZ induced a concentration-dependent toxic effect on the test systems. It also exerted cytotoxicity in fibroblasts and human gastric cells. In addition, TRZ showed mutagenic effects on the *A. cepa* test system. However, its toxicogenic effects may not relate to the oxidizing activity, which was confirmed by the *S. cerevisiae* test model.

**Conclusion:**

Taken together, TRZ exerted toxicogenic effects on the test systems. Therefore, it may be harmful to health, especially its prolonged use may trigger carcinogenesis.

## Background

Food additives are strategically important during food production. However, incipient data are available regarding the possible toxicological risks caused by the frequent ingestion of these substances [1]. Different studies also suggest the toxic effects of food additives, including acute or chronic toxicity, triggering allergic processes, neurobehavioral alterations, cellular neoplasms, and so on [2, 3].

Artificial dyes belong to the class of food additives that have been the subject of much criticism among researchers, since their use in food is justified only by the customer’s eating habits [4]. Several studies have shown that the dyes are genotoxic food additives [5], especially those belonging to the “azo” group. This “azo” group dyes are nitrous derivatives, capable of causing hypersensitivity reactions and have been the focus of mutagenesis and carcinogenesis studies for producing aromatic amines and sulfanilic acid after being metabolized by the intestinal microflora [6].

“Tartrazine” (TRZ) is a type of artificial dye containing an “azo” group, widely studied by toxicologists and allergists, since it is related to several adverse reactions, such as urticaria, asthma, nausea, eczema, bronchitis, rhinitis, bronchospasm, and headache [7]. Nevertheless, it is one of the most commonly used dyes in foods, being allowed in several countries in the world [8]. TRZ is present in daily consumed products like soft and sports drinks, flavored potatoes, sauces, ice cream, jellies, and chewing gum [9]. In developing countries, TRZ is used as a low-cost cooking alternative for the use of saffron [10]. Moreover, it is also found in many non-food products, such as soaps, cosmetics, shampoos, vitamins, and medications [11].

Several dyes have been evaluated for their toxicogenic effects, and many of them showed significant cytotoxicity and mutagenicity, such as “Light blue” and “Allura red” (red 40) [12], “Amaranth” [13],“Green S” [14], and TRZ [15–17]. However, the toxicogenetic effects of TRZ remain inconclusive since in some *in vivo* studies, where animals received TRZ at different doses, no cytotoxic changes were observed in tissues and organs. However, the development of neoplastic abnormalities was observed in many cases [18].

Therefore, considering the use of TRZ worldwide, mainly by children, and the lack of conclusive studies concerning the toxicogenic profile of this dye, the present study aims to evaluate the cytotoxic and mutagenic potential of TRZ in different eukaryotic models.

## Methods

### Tartrazine (TRZ) and preparation of test concentrations

The TRZ powder (CAS 1934-21-0, purity ≥85 %) purchased from Sigma-Aldrich (St. Louis, MO, USA) was diluted in distilled water at 10 mg/mL as a stock solution. According to the Joint Expert Committee on Food Additives [19], the present study used the following concentrations of TRZ: 100, 200, and 400 μg/mL for the evaluation of cytotoxic and mutagenic effects in *Allium cepa* and *Saccharomyces cerevisiae* strains. For the tests involving serial dilutions, MTT and *Artemia salina* TRZ at 1.5 to 100 and 31.25 to 1000 μg/mL were used, respectively.

### Cell culture

Normal stomach cell line (MN01) and normal human fibroblast cell line (FGH) acquired from Banco de Células do Rio de Janeiro (BCRJ, Brazil). This primary cell culture was obtained by tripsin digestion of the entire rat heart followed by collagenase type II treatment. The cell lines were cultured in Dulbecco's Modified Eagle's medium (DMEM) supplemented with 10% fetal bovine serum (FBS), 1% antibiotics (penicillin/streptomycin) and placed in humidified air at 37 °C with a 5% CO_2_ atmosphere. A Trypsin/EDTA 0.25% solution was used to detach and harvest the cells before the experiments. The medium was changed after 48 hours of culture.

### Cell viability assay

The non-tumoral gastric mucosal (MN01) cells were seeded in 96-well plates with 3×10^3^ cells/well, and further incubated in triplicate with TRZ at concentrations ranging from 1.5 to 100 μg/mL for 72 h at 5 % CO_2_ and 37 °C. Doxorubicin (16 μM) and the vehicle were used as positive and negative controls, respectively. After incubation with the drugs, the cell supernatant was removed, and then 100 μL MTT solution (0.5 mg/mL) was added for an additional 3 h in the same conditions described above. Formazan crystals were dissolved in dimethyl sulfoxide (DMSO) (100 μL/well) for 10 min in a shaker, and the absorbance was recorded in a microplate spectrophotometer (BioTek, Winooski-VT, USA) at 550 nm [20].

Cell viability was calculated by the percentage of cell viability inhibition *×* log of TRZ concentration, and the half maximal inhibitory concentration (IC_50_) was determined at 95% confidence intervals by using non-linear regression analysis (GraphPad Prism v. 7.0, San Diego, California, US, 2018).

### Brine shrimp lethality bioassay (BSLB)

Cysts of *Artemia salina*, acquired in the central market of Teresina (Piaui), Brazil, were used to evaluate the toxicity of TRZ. According to Meyer et al. [21] with modifications, the *A. saline* cysts were incubated in a beaker containing a 50:50 mixture of saline (artificial sea water: 23.0 g NaCl, 11.0 g MgCl_2_.6H_2_O, 4 g Na_2_SO_4_, 1.3 g CaCl_2_.2H_2_O, 0.7 g KCl, in 1 L distilled water at pH 8.5 (adjusted by using 1 N Na_2_CO_3_) and mineral water under constant aeration for 48 h at 27 ± 3 °C. After incubation, the live nauplii free from microcrustacean shells were collected from the lighter portion of the incubation chamber and are used for this assay. Ten (10) nauplii were placed into each test tube containing 4.5 mL of the saline solution. The experiment was performed by serial dilutions of TRZ at 31.25 to 1000 μg/mL. In each experiment, 0.5 mL of the test sample was added to 4.5 mL of saline solution, and the mortality of *A. salina* was recorded after 48 h of exposure time.

Toxicity was based on the toxicity scales of Collins and McLaughlin [22]. According to this scale, lethal concentration (LC_50_) values >1000, 500 to 1000, within 100-500, and <100 μg/mL were considered non-toxic, low toxic, moderately toxic, and highly toxic, respectively.

### *Allium cepa* test

In order to verify the cytotoxic and mutagenic effects of TRZ, the *Allium cepa* test was performed by using small onions of uniform size from the same origin, non-germinated and healthy. The onions were placed in small glass containers (Capacity: 10-15 mL) filled with water for rooting and kept in a dark room at 27 ± 2 °C. Onions with satisfactory root growth were placed in the treatment solutions, divided as: T1 - negative control (NC), where the roots of the bulbs were treated with distilled water alone; T2 - TRZ 400 µg/mL; T3 - TRZ 200 µg/mL ; T4 - TRZ 100 µg/mL; and T5 - positive control (PC), treated with copper sulfate (0,006 µg/mL). The onions were treated for up to 72 hours. The growth of two selected roots in each bulb was measured by a scale in mm. The acquisition of the images was performed using a biological planarchromatic infinity-optics microscope (Bioptika brand and model B605) with 10-megapixel color CMOS digital camera. The objective of 40 was used, thus using a resolution of 400X. The acquisition software was IS Capture 2.5, version 2.5.1547.4007.

### Cytogenetic analysis in *A. cepa*

The slides (3 per onion) were prepared following the method of Guerra and Souza [23] and analyzed by a trinocular biological microscope, the Bioptika B20 at 40x magnification. For each onion, 1*×*10^3^ cells were analyzed, totaling 5×10^3^ cells for each treatment. Cells were observed during the cell division phases of interphase, prophase, metaphase, anaphase, and telophase. The number of cells in interphase and mitosis of each treatment and time of exposure was calculated, and then the mitotic index (MI) was determined for cytotoxic evaluation. TRZ mutagenicity was performed by counting the number of cells with chromosomal alterations (CA).

$$\mathrm{Mitotic}\;\mathrm{index}\;\mathrm{calculation}\;\mathrm{formula}:\;(\mathrm{Cells}\;\mathrm{in}\;\mathrm{mitosis}\;\div\;\mathrm{Total}\;\mathrm{number}\;\mathrm{of}\;\mathrm{cells})\;\times\;100$$

### *Saccharomyces cerevisiae* strains

Six different strains of *S. cerevisiae* were used to evaluate the oxidative effect of TRZ. The wild-type strain used in this assay has no mutation in the defense enzymes against oxidative substances, while the other five strains selected have defects in at least one antioxidative enzyme. The strains were provided by researcher João Antônio Pegas Henriques from the Biosciences Institute of the Federal University of Rio Grande do Sul - BRAZIL. The EG118 strain is mutated in the cytoplasmic superoxide dismutase enzyme (CuZnSOD - *SOD1* gene product); EG110 is mutated in mitochondrial SOD (MnSOD - *SOD2* gene product); EG133 has a two-enzyme mutation to *SOD1* and *SOD2*; EG223 mutated in *CAT1* and EG mutated in *SOD1* and *CAT1* (Table 1).

**Table 1:** *Saccharomyces cerevisiae* strains used in the study

**Strains**	**Genotype**	**Origin**
EG103 (SODWT)	MATa leu2-3,112 trp1-289 ura3-52 GAL+	Edith Gralla, L Angeles
EG118 (Sod1Δ)	sod1:URA3 all other markers as EG103	
EG110 (Sod2Δ)	sod2:TRP1 all other markers as EG103	
EG133 (Sod1ΔSod2Δ)	sod1:URA3 sod2:TRP1 double mutant all other markers as EG103	
EG223 (Cat1Δ)	EG103, except Cat1: TRP1	
EG (Sod1ΔCat1Δ)	EG103, except Sod1: URA3 and Cat1: TRP1	

Strains were kindly provided by the research group in Genetic Toxicology at the Federal University of Rio Grande do Sul (UFRGS)

### Oxidative assay in *S. cerevisiae*

All experiments were performed using the central disk test in *S. cerevisiae* culture, exposed to different concentrations of TRZ (100 - 400 µg/mL). The strains were grown in YEL medium (0.5 % yeast extract, 2 % bacto-peptone, and 2 % glucose) and kept at 28 °C in a shaker until reaching the stationary growth stage, according to Oliveira et al. [12]. Cell suspensions were seeded from the center to the margin of Petri dishes, on both sides, containing in their center a disc of sterile filter paper, to which 10 μL of TRZ was added at each concentration. During the experiments, H_2_O_2_ (10 mM) and saline solution (0.9 %) were used as positive control (PC) and negative control (NC) groups, respectively.

After 48 h of incubation at 34 ºC, the growth inhibition halos (in mm) were measured from the margin of the filter paper disk until the beginning of the strain’s growth. The values were organized and subsequently analyzed, ranging from 0 (complete growth) to 40 mm (absence of growth), corresponding to the measurement of the Petri dish radius. All tests were performed in duplicate.

### Statistical analysis

ANOVA one-way followed by Dunnett’s test for MTT and Bonferroni’s test for the other methodologies was applied by considering *p* <0.05. The IC_50_ values were obtained by plotting the Hill equation: f = Min + (Max-Min) / (1+ (IC_50_ / [drug] n), where Max and Min represent the maximum and minimum values, respectively; IC_50_ is the effective concentration of a substance that kills 50% of the cells evaluated; and n is the "Hill" coefficient of the substance. GraphPad Prism Software v.7.0 (San Diego, California, US) was used for the analysis.

## Results

### Cytotoxicity in normal stomach cells (MTT assay)

**Fig. 1:**
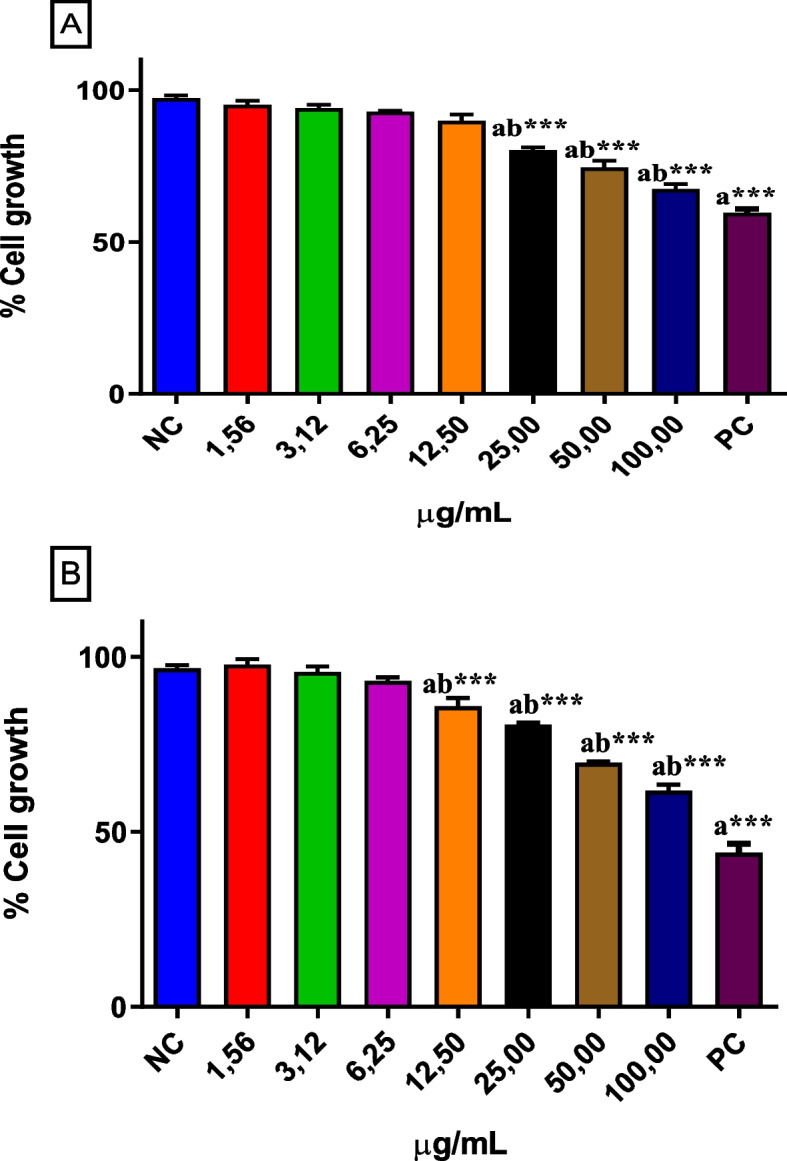
Viability of normal stomach cell line (MN01) (Figure 1**A**) and normal human fibroblast cell line (FGH) (Figure 1**B**) exposed to different concentrations of tartrazine (1.56 - 100 μg/mL) [Values ​​are mean ± SD; ^a^compared to the NC and ^b^compared to the PC group; ***p <0.001. ANOVA one-way, followed by the Tukey post-test]

The TRZ dye showed significant cytotoxic effects on the MN01 from 25 μg/mL to the highest test concentration (100 μg/mL) for MN01 (normal stomach human cell line) and from 12.5 to 100 μg/mL for FGH (normal fibroblast human cell line), since cell viability was reduced compared to the NC group (*p* >0.001). The PC (doxorubicin) at 16 μM reduced cell viability by about 40% and 62% when compared to the NC group, respectively (Fig. 1).

### Toxic effects of TRZ on *A. salina*

**Fig. 2:**
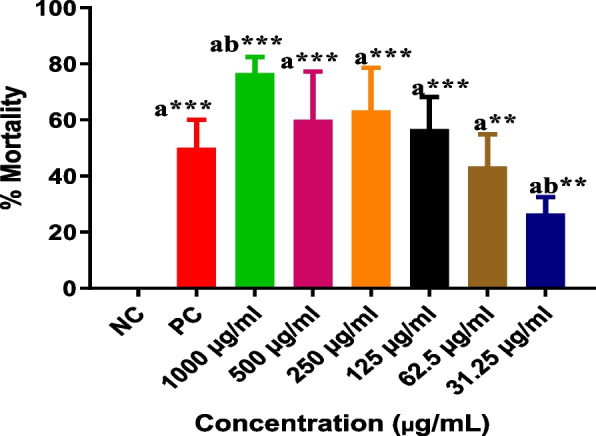
Toxic effects of tartrazine dye on *Artemia salina* at different concentrations (31.25 – 1000 μg/mL) after 48 h of incubation [Values ​​are mean ± SD, ^a^compared to the NC and ^b^compared to the PC group (K_2_Cr_2_O_7_, 16 µM); **p<0.01; ***p<0.001. ANOVA one-way, followed by the Tukey post-test. Each concentration was tested in triplicate (10 nauplii/tube)]

TRZ showed significant toxicity after 48 h on the *A. salina* system, even at the smallest concentration tested (31.25 μg/mL), which caused about 30% death of nauplii (*p* <0.01). The LC_50_ value of TRZ was 221.5 μg/mL, which was higher than the value recommended by the Brazilian Health Regulatory Agency [24] and [19]. By the toxicity classification of Collins and McLaughlin [22], TRZ presented moderate toxicity (100 to 500 μg/mL) (Fig. 2).

### Toxic and cytotoxic effects on *A. cepa*

**Fig. 3:**
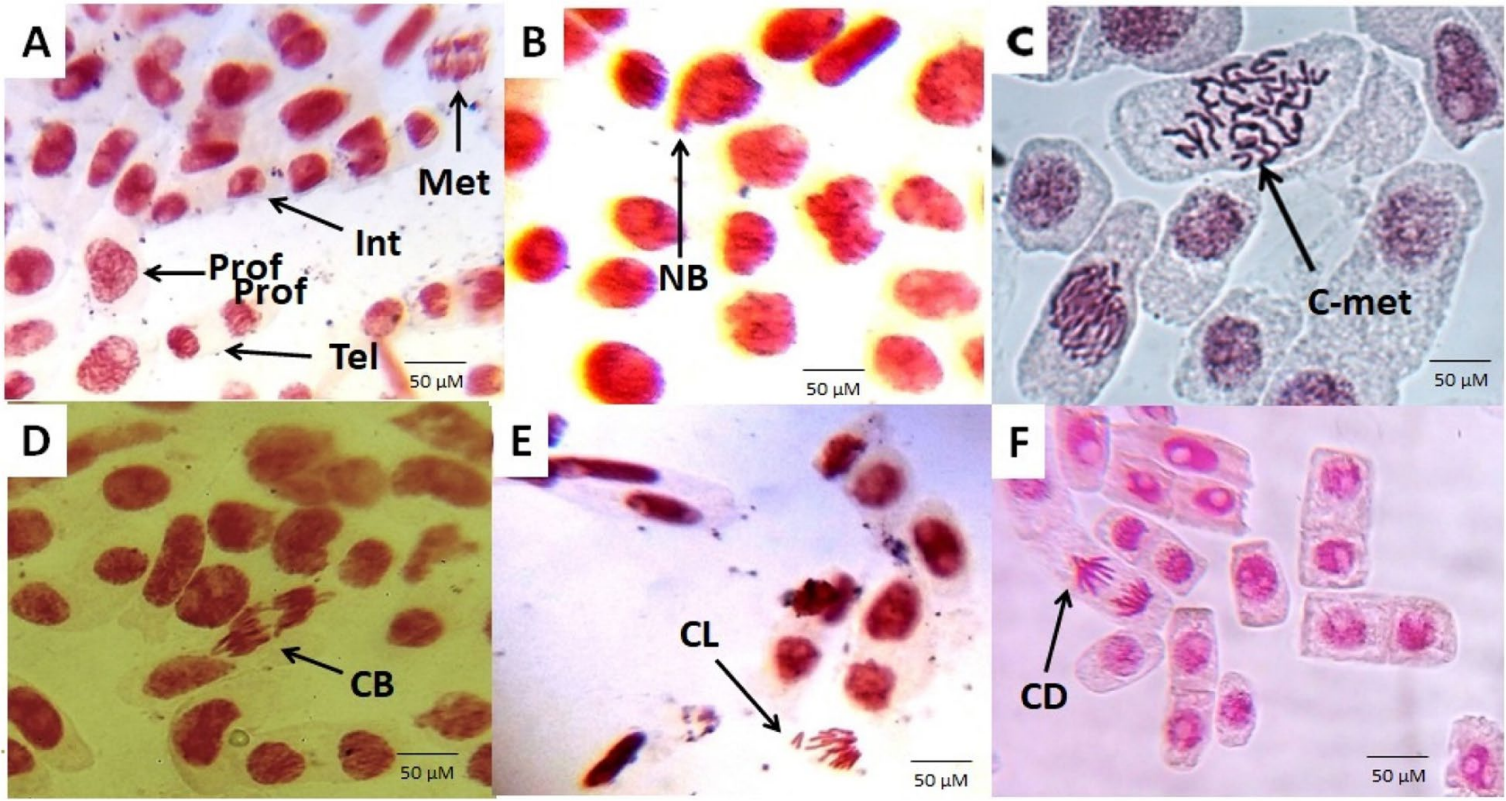
Photomicrographs of meristematic *Allium cepa* cells treated with tartrazine [Cells were colored by acetic orcein and observed at 400x by optical microscopy. **a** Cells at different cell cycle phases (Int – Interphase; Prof – Prophase; Met – Metaphase; Tel – Telophase) in negative control; **b** NB (Bud nuclear) in ​​cells treated with the positive control; **c** C-met (colchicine metaphase) in cells treated with 100 µg/mL of TRZ; **d** CB (chromosome bridge) in cells treated with 200 µg/mL of TRZ; **e**: CL (chromosomal loss) in cells treated with 400 µg/mL of TRZ; **f** CD (chromosome delay) in cells treated with 400 µg/mL of TRZ

The toxicogenetic study of TRZ was performed by analyzing the root growth (RG), the mitotic index (MI) and chromosomal aberration (CA) parameters (Fig. 3). For all test concentrations and exposure times (ET) of TRZ, significant differences were found for RG and MI when compared to the NC, indicating toxic and cytotoxic effects of this dye (Table 2). When compared to the PC group, TRZ at all concentrations showed less toxic effects for both variables (RG and MI). TRZ was found to promote cell division arrest in *A. cepa* meristematic cells, which was confirmed by a higher number of interphase cells rather than the cells in cellular division phases (*p* <0.05).

**Table 2:** Cytogenetic profile of TRZ in meristematic cells of *Allium cepa*

**Treatments**	**Concentrations**	**ET (h)**	**RG (mm)**	**Cell cycle phases**					**MI (%)**
				***Interphase***	***Prophase***	***Metaphase***	***Anaphase***	***Telophase***	
NC	-	24	25.5 ± 2.8	476.2 ± 12.3	423.8 ± 7.85	41.0 ± 1.6	30.7 ± 2.6	28.2 ± 2.8	52.3 ±1.51
PC (ug/mL)	6		11.7 ± 0.5^a^	842.1 ± 12.2^a^	100.0 ± 13.9^a^	26.75 ± 4.0^a^	17.2 ± 2.5	14.0 ± 1.9^a^	15.8 ± 1.1^a^
TRZ (µg/mL)	400		15.0 ± 1.4^ab^	703.75 ± 56.3^ab^	259.0 ± 68.1^ab^	10.75 ± 0.9^ab^	12.5 ± 0.5^a^	14.0 ± 2.1^a^	29.6 ± 7.1^ab^
	200		16.0 ± 0.8^ab^	665.1 ± 73.9^ab^	297.2 ± 56.4^ab^	14.8 ± 1.4^a^	10.4 ± 1.7^ab^	12.5 ± 1.9^a^	33.4 ± 7.7^ab^
	100		16.1 ± 0.6^ab^	700.1 ± 48.7^ab^	252.2 ± 34.4^ab^	16.6 ± 1.9^a^	15.6 ± 1.0^a^	15.2 ± 0.9^a^	30.1 ± 6.5^ab^
NC	-	72	36.2 ± 3.5	487.5 ± 12.3	412.5 ± 7.8	41.0 ± 1.63	30.7 ± 2.6	28.2 ± 2.8	51.2 ± 1.2
PC (µg/mL )	6		14.5 ± 0.5^a^	873.0 ± 15.1^a^	65.0 ± 15.7^a^	29.7 ± 6.0	19.2 ± 3.6	14.0 ± 2.4^a^	12.8 ± 1.3^a^
TRZ (µg/mL)	400		19.8 ± 2.3^ab^	652.8 ±74.3^ab^	316.0 ± 58.1^ab^	11.5 ± 0.9^a^	10.5 ± 0.5^a^	9.2 ± 2.1^a^	34.7 ± 10.5^ab^
	200		18.5 ± 1.2^ab^	678.2 ± 63.9^ab^	280.4 ± 46.4^ab^	16.8 ± 1.9^a^	13.2 ± 2.1^ab^	11.4 ± 2.9^a^	32.1 ± 5.6^ab^
	100		23.2 ± 0.9^ab^	648.0 ± 88.7^ab^	300.7 ± 34.4^ab^	20.5 ± 2.0^a^	15.8 ± 1.7^a^	14.2 ± 1.1^a^	35.1 ± 8.8^ab^

Values ​​are mean ± SD. ANOVA one-way, followed by the Tukey post-test. ^a^compared to the NC and ^b^compared to the PC group; *p* < 0.05; *t(exposure)/h* Exposure time, *RG* Root growth in mm, *MI* Mitotic index, *TRZ* Tartrazine, *NC* Vehicle (negative control), *PC* Positive control (CuSO_4_.5H_2_O)

### Mutagenic effects on *A. cepa*

Regarding mutagenic evaluation, TRZ at all test concentrations (100 - 400 µg/mL) induced mutagenicity in *A. cepa* cells at 24 and 72 h exposure times (*p* <0.05). The TRZ showed a significant clastogenic capacity by increasing the micronuclei (MN) formation at the highest concentration (400 µg/mL). TRZ also demonstrated the capacity to cause disturbances in the mitotic spindle by increasing c-metaphase damage (Table 3).

**Table 3:** Mutagenic effects of tartrazine in meristematic cells of *Allium cepa*

**Treatments**	**Concentrations**	**ET (h)**	**Chromosomal alterations (CA)**					**Total CA**
			***Micronuclei***	***c-metaphases***	***Bridges***	***Losses***	***Delays***	
NC	-	24	0.2 ± 0.5	0.2 ± 0.5	0.25 ± 0.5	0.0 ± 0.0	0.75 ± 0.5	1.40 ± 0.5
PC (ug/mL)	0.006 mg/ml		3.8 ± 0.9^a^	4.5 ± 0.5^a^	10.2 ± 0.9^a^	5.25 ± 2.62^a^	10.5 ± 1.29^a^	34.25 ± 4.7^a^
TRZ (µg/mL)	400 µg/mL		3.0 ± 0.9^a^	6 ± 4.1^a^	1.5 ± 1.2^b^	0.75 ± 0.5^b^	2.5 ±1.29^b^	15.04 ± 4.2^ab^
	200 µg/mL		2.3 ± 0.9	6 ± 4.1^a^	1.0 ± 2.0^b^	7.0 ± 7.52^a^	0.5 ± 1.0^b^	16.75 ± 4.3^ab^
	100 µg/mL		2.2 ± 2.0	4.5 ± 1.7^a^	1.0 ± 1.41^b^	0.75 ± 0.95^b^	0.75 ± 0.95^b^	9.2 ± 3.5^ab^
NC	-	72	0.2 ± 0.5	0.15 ± 0.5	0.0 ± 0.0	0.2 ± 0.5	0.4 ± 0.3	0.9 ± 0.3
PC (µg/mL )	0.006 mg/ml		4.2 ± 0.7^a^	4.9 ± 0.4^a^	12.3 ± 1.2^a^	7.2 ± 1.9^a^	11.4 ± 0.9^a^	40.05 ± 4.9^a^
TRZ (µg/mL)	400 µg/mL		4.0 ± 3.1^a^	2.5 ± 1.2	2 ± 1.4^b^	0.25 ± 0.5^b^	2.0 ± 2.4^b^	10.75 ± 2.5^ab^
	200 µg/mL		2.7 ± 1.2	4.25 ± 3.0^a^	0.5 ± 0.5^b^	0.75 ± 0.5^b^	0.7 ± 1.5^b^	9.0 ± 2.2^ab^
	100 µg/mL		2.0 ± 1.41	3.5 ± 1.9^a^	1.0 ± 1.1^b^	1.0 ± 2.0^b^	1.2 ± 1.8^b^	8.75 ± 1.2^ab^

Values ​​are mean ± SD; ANOVA one-way, followed by the Tukey post-test; *p*< 0.05, ^a^compared to the NC and ^b^compared to the PC group; *ET* Exposure time, *CA* Chromosomic alterations, *TRZ* Tartrazine, *NC* Vehicle (negative control), *PC* Positive control (CuSO_4_.5H_2_O)

### Oxidative effects on *S. cerevisiae*

TRZ at any test concentration did not induce an oxidizing effect on the yeast strains. Thus, the cytotoxic and mutagenic effects observed in the present study are probably not related to oxidative stress pathways (Table 4).

**Table 4:** Oxidizing effects of tartrazine at different concentrations using mutant *Saccharomyces cerevisiae* strains

**Test strains**	**Treatments**				
	**NC**	**PC**	**TRZ**		
	***Saline***	***H***_***2***_ ***O***_***2***_	***400 µg/mL***	***200 µg/mL***	***100 µg/mL***
SODWT	0.75 ± 0.50	14.35 ± 0.25ª	0.00 ± 0.00	0.30 ± 0.50	0.00 ± 0.00
Sod1*∆*	1.50 ± 0.57	14.73 ± 2.28^a^	1.00 ± 0.80	0.80 ± 0.80	0.00 ± 0.00
*Sod2∆*	1.25 ± 0.50	13.82 ± 0.45^a^	0.50 ± 0.57	0.75 ± 0.89	0.00 ± 0.00
*Sod1Sod2∆*	2.00 ± 0.81	11.35 ± 1.01^a^	0.00 ± 0.00	0.00 ± 0.00	0.00 ± 0.00
*Cat∆*	1.25 ± 0.50	15.10 ± 0.70^a^	1.00 ± 0.80	0.75 ± 0.50	0.00 ± 0.00
*Sod1Cat*	1.50 ± 0.57	12.37± 0.22^a^	0.10± 1.15	0.75± 0.90	0.00 ± 0.00

Values ​​are mean ± SD of inhibition halos measured in Petri dishes (0-40 mm). ANOVA one-way followed by the Tukey post-test; ^a^ *p*<0.001 compared to the NC (saline),^b^ *p*<0.001 compared to the PC (H_2_O_2_); *TRZ* Tartrazine

## Discussion

Artificial dyes are commonly used to improve foods’ appearance. However, some of these dyes appear in the medical literature as potential inducers of various human diseases [25]. TRZ was recently evaluated for its safety as a food additive by the Food and Agriculture Organization (FAO)/World Health Organization (WHO) Expert Committee on Food Additives (JECFA) during the 2016 meeting [26]. Previously, TRZ had been evaluated by the European Food Safety Authority (EFSA) in 2009 and 2013 [27, 28]. Until 2016, JECFA had established an acceptable daily intake (ADI) of 0 - 7 mg/kg body weight (bp)/day for TRZ, based on a NOAEL dose equivalent to 750 mg/kg/day derived from a chronic toxicity study in rats. After 2016, JECFA increased the acceptable daily intake (ADI) to 0 - 10 mg/kg/day [26], based on the absence of any convincing evidence of adverse effects at the highest dose levels tested (1000 mg/kg/day) for long-term studies in reproduction and development of animals.

However, studies have sought a better characterization of the adverse effects that TRZ may trigger in the human body, especially at the molecular level. Various studies have shown consistent data on TRZ that it can induce systemic toxicity [11], can affect metabolism and body development [29], interact with the hormone receptors [30], and cellular DNA [31]. In addition, TRZ is able to promote allergy [29] and induce genotoxic and cytotoxic effects in animals [32].

The MTT test showed significant cytotoxicity on both human stomach and rat fibroblast cell lines. Previous studies have evaluated TRZ cytotoxic effects *in vitro* and *in vivo* models [33, 32]. Balta et al. [33] evaluated TRZ cytotoxicity on the liver, kidney, spleen, and brain of albino Wistar rats, demonstrating that TRZ significantly increased kidney and liver weight while reducing spleen weight in comparison to the group control. Moreover, a histopathological assay showed that TRZ produced lesions in the kidney, spleen, and liver of all rodents. Tartrazine promoted histopathological changes, causing significant liver tissue damage and changes in blood parameters.

In our study, we suggest the TRZ cytotoxic mechanism involved is related to interfering in the cell cycle, since most *Allium cepa* meristematic cells were found in interphase, which shows its ability to block cell division. This directly affects mitotic division (MI) rates and root growth, which agrees with the studies by Mpountoukas et al. [32].

Another event that compromises the cell cycle is related to DNA damage. Our study observed a correlation between decreased root growth and mitotic rate in relation to chromosomal aberrations. According to Glaser and Stopper [34], DNA damage leads to cell cycle delays and multipolar mitotic spindle formation. Thus, the present study suggests that TRZ mutagenic effects are explained by its clastogenic capacity through micronuclei (MN) formation, which is the more effective and simpler indicator of cell damage [35],as well as the ability to disrupt the mitotic spindle that forms c-metaphase. In c-metaphase, the nuclear spindle is completely inactivated, which means that no equatorial plate becomes organized and, consequently, centromere division is delayed or even prevented [36]. These mechanisms are corroborated by Soares et al. [17], in which TRZ promoted a direct effect on DNA in different eukaryotic test systems, including human gastrointestinal cells [37].

Supporting the present study, the TRZ dye showed cytotoxic and mutagenic effects in the studies by Mpountoukas et al. [32]. They described the genotoxic effects of TRZ dyes, Amaranth, and Erythrosine B27 on peripheral blood cells, demonstrating that TRZ can affect mitotic cell division rates at higher concentrations (4 and 8 mM). This study also suggests that TRZ has the ability to bind to the DNA structure.

Kashanian and Zeidali [30] used the DNA from calf thymus cells to visualize the binding properties of TRZ (10 nM) and consequently its genetic adverse effects. Their study demonstrated that DNA-TRZ interaction affected the DNA helical structure, which was easier when DNA was in denatured form.

Among the various enzymatic systems responsible for metabolic processes, the major biotransformation routes of azo dyes, such as TRZ, involve the cytochrome P-450 complex (CYP). CYP enzymes belong to a superfamily of heme proteins that are present in all living organisms. They are involved in the metabolism of a wide variety of chemical compounds and have the ability to catalyze oxidative and reductive reactions of xenobiotics [38]. The reduction of azo dyes can occur in the liver *via* CYP enzymes, generating products with carcinogenic properties, such as aromatic amines. The toxicity and carcinogenicity of certain azo dyes in mammals is also discussed following biotransformation reactions catalyzed by enzymatic reactions, including those catalyzed by azo reductase present in the mammalian intestine. The products generated may be more or less toxic than the original molecule [39].

Atlı Şekeroğlu et al. [15] demonstrated that TRZ (625, 1250, and 2500 µg/mL) and its metabolites have cytotoxic activity in human lymphocyte cell culture in the presence and absence of a metabolic activator (mix S9). MI was at higher concentration due to a significant decrease in MI in the absence of mix S9 when compared to the control group. Furthermore, at higher concentrations, TRZ and its metabolites significantly increased MN formation, CA, and aberrant cells in the presence and absence of mixed S9.

Soares et al. [17], evaluating *in vitro* cytotoxicity, genotoxicity, and DNA repair in human lymphocytes exposed to TRZ, demonstrated that this additive has no cytotoxic effect. However, TRZ showed significant genotoxic effects at all concentrations tested (0.25 - 64.0 mM). Although most DNA damage was repaired, some damage was significantly greater than the PC after 24 h of DNA repair. These preliminary data demonstrate that TRZ could be harmful to health and its prolonged use may trigger carcinogenesis.

Bastaki et al. [18] recently assessed TRZ (25, 500 or 2000 mg/kg) genotoxicity by using *in vivo* models and showed no genotoxic activity. On the other hand, Sasaki et al. [37] used the comet assay to evaluate the DNA damage of various organs caused by the consumption of food additives in mice, and found that the dyes had higher genotoxic effects. TRZ has been shown to cause damage to the colon cells, even at low doses (10 mg/kg) close to ADI (7.5 mg/kg). However, the authors stated that the toxicity of these substances may vary among animals.

In the *A. salina* toxicity assay, TRZ was moderately toxic, although the study by Imane et al. [40] showed no significant toxicity for the same in vivo test, except when they evaluated the main TRZ metabolite, sulfanilic acid, where they found mild toxicity. Conversely, Atlı Şekeroğlu et al. [15] using TRZ metabolites demonstrated genotoxic activity in human lymphocyte cells. Although there are controversial results, the present study also showed the TRZ toxic effect in the *A. cepa* test with root growth reduction. This shows that TRZ toxicity has been demonstrated in different organisms (animals and plants). More sensitive *in vitro* and *in vivo* studies using different models are needed to evaluate TRZ genotoxic and mutagenic effects. TRZ induces DNA damage, which is one of the major causes of cancer in animals. Therefore, the successive accumulation of damage caused by regular intake of food containing TRZ can lead to DNA mutations and, ultimately, the onset of diseases such as cancer. 

## Conclusion

The artificial dye TRZ showed toxic, cytotoxic, and mutagenic effects on plant, animal, and human cells. Our results point out that TRZ has a moderate toxic effect in different pre-clinical test models. TRZ is clastogenic and causes mitotic spindle disorders. However, its toxicogenic effects were not related to oxidizing activity. These data demonstrated that TRZ may be harmful to health and its prolonged use is thought to trigger carcinogenesis. Our findings support the need for better food additive inspections by regulatory agencies, as well as the elimination of tartrazine as a food additive because it provides no nutritional benefit.

## Data Availability

The datasets used and analyzed during the current study are available from the corresponding author upon reasonable request.
